# Editorial: Research advances in intestinal diseases and related diarrhea in animal production

**DOI:** 10.3389/fvets.2023.1201231

**Published:** 2023-05-04

**Authors:** Wenjuan Du, Xianghuang Wang, Le Xu, Xihong Zhou, Deguang Song, Qingbiao Xu

**Affiliations:** ^1^College of Animal Sciences and Technology, Huazhong Agricultural University, Wuhan, China; ^2^Key Laboratory of Agro-ecological Processes in Subtropical Region, Institute of Subtropical Agriculture, Chinese Academy of Sciences, Changsha, China; ^3^Department of Immunobiology, Yale University School of Medicine, New Haven, CT, United States

**Keywords:** intestinal diseases, diarrhea, microbiota, inflammation, nutrition

Animal diarrhea is a prevalent issue in animal production globally and causes significant economic losses to animal husbandry. Typically, the occurrence of diarrhea is closely related to imbalances in intestinal microbiota. Animals with diarrhea have an altered intestinal microbiota balance, and infection by pathogens can result in a diarrhea phenotype (1). Diarrhea mainly occurs in newborn young animals. Because the digestive tract of young animals is not fully developed, they are vulnerable to infection by pathogens. The healthy development of young animals is the key to improving the growth and development of adult animals. Therefore, it is crucial to give special attention to animal diarrhea and take effective prevention and treatment measures. The aim of this Research Topic is to raise awareness about animal diarrhea, gather the latest research findings, and employ nutrition intervention strategies, such as adding bioactive substances, natural plant extracts, or feed additives, to reduce the incidence of animal diarrhea.

Neonatal calf diarrhea is a globally recognized disease in the cattle industry. With its high incidence rate, mortality, growth retardation, treatment costs, and severe long-term consequences, it has caused significant economic losses (2). Antibiotics have been commonly used to treat calf diarrhea and enhance livestock growth. However, the use of antibiotics in animal husbandry has numerous side effects. The main issues include the emergence of drug-resistant bacteria and the presence of antibiotic residues in meat (3). The first study by Zong et al. explored the effect of the oral milk-derived bioactive tripeptide, VPP (Val-Pro-Pro), on the degree of diarrhea and intestinal inflammation in pre-weaning Holstein calves. In this study, 18 calves with similar birth dates, weights, and genetic backgrounds were selected. The calves were randomly assigned to two groups (*n* = 9): the control group (PBS solution, 50 mL/d) and the VPP group (VPP solution, 100 mg/kg BW/d). The test lasted for 17 days. The findings revealed that oral administration of VPP resulted in decreased levels of pro-inflammatory factors in the blood, increased concentrations of short-chain fatty acids (namely n-butyric and isovaleric acids) in stool, and altered the intestinal microbiota of calves. Consequently, VPP reduced the severity of calf diarrhea prior to weaning. In this study, the author found that administering VPP to calves before weaning was a practical strategy to reduce the degree of intestinal inflammation and diarrhea in calves, which is essential to improve the future production performance of calves and cultivate excellent reserve cattle.

Rumen bloating is a common digestive system disease in ruminants, characterized by gas accumulation in the rumen and reticular rumen, and may damage digestive and respiratory functions (including diarrhea). Changes in modern feeding methods have led to an increase in the incidence of abdominal bloating in grazing and enclosed ruminants (4). The second paper written by Wang et al. explores ways to treat rumen swelling through nutritional regulation. The results showed that supplementation with concentrated tannins and other additives can effectively prevent and treat rumen swelling caused by feeding with high-concentration diets.

Modern intensive breeding systems frequently utilize early weaning technology to enhance sow productivity, leading to an increased annual litter size for sows (5). However, the stress caused by early weaning will lead to diarrhea, which will increase the mortality of piglets and reduce growth performance (6). Enterotoxigenic *Escherichia coli* (ETEC) is the primary cause of post-weaning diarrhea (PWD) in piglets (7). The third paper written by Jerez-Bogota et al. conducted a series of experiments on weaned piglets. The study investigated the effects of dietary supplements such as garlic, apple pomace, or black currant on infection indicators and fecal microbiota in piglets exposed to ETEC F18 attacks. In this experiment, 32 piglets aged 7 weeks were randomly divided into four groups: non-challenge (NC), ETEC-challenge (PC), ETEC-challenge accepting garlic and apple pomace (GA), and ETEC challenge accepting garlic and blackcurrant (GB). The results showed that the incidence of diarrhea in piglets in the GA and GB groups was significantly lower than that in ETEC-infected piglets, and the Prevotella and Lactobacillus in the GB group were more abundant than those in the PC group. In summary, dietary supplementation with GA and GB limits ETEC proliferation, reduces PWD, and beneficially affects the diversity, composition, and stability of fecal microbiota.

The fourth article written by Bonetti et al. conducted a similar study *in vitro*. The author explored the protective effects of a group of essential oils and natural extracts on intestinal Caco-2 cells infected with Escherichia coli F4 infection. The experimental results indicated that thyme essential oil (ThyEO) and grape seed extract (GSE) can effectively protect the epithelial integrity of Caco-2 cells under the infection of Escherichia coli F4; GSE effectively reduces the inflammatory response, and ThyEO and ginger essential oil significantly reduce the adhesion of Escherichia coli F4. These *in vitro* and *in vivo* experimental results support the use of dietary supplements and plant extracts to manage PWD in piglets.

This Research Topic strengthens our understanding of various strategies for preventing post-weaning diarrhea in young animals and provides new ideas for further research in this field. Although these four articles have proposed feeding strategies to alleviate gastrointestinal diseases in animals, there are still some areas of knowledge that need to be explored. In order to optimize nutritional regulation and promote the healthy development of young animals, future research should focus on exploring the role of nutrition in improving the intestinal health of pre-weaning calves and piglets. It is crucial to explore potential mechanisms for alleviating gastrointestinal diseases in animals through advanced technologies such as metagenomics and metabolomics to identify new biomarkers. In addition, future research should study the long-term impact of nutrition control measures on the growth and development of young animals in order to develop more effective nutrient management strategies, prevent digestive tract problems in animals, and promote the development of the livestock industry.

## Author contributions

All authors listed have made a substantial, direct, and intellectual contribution to the work and approved it for publication.
